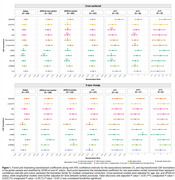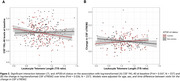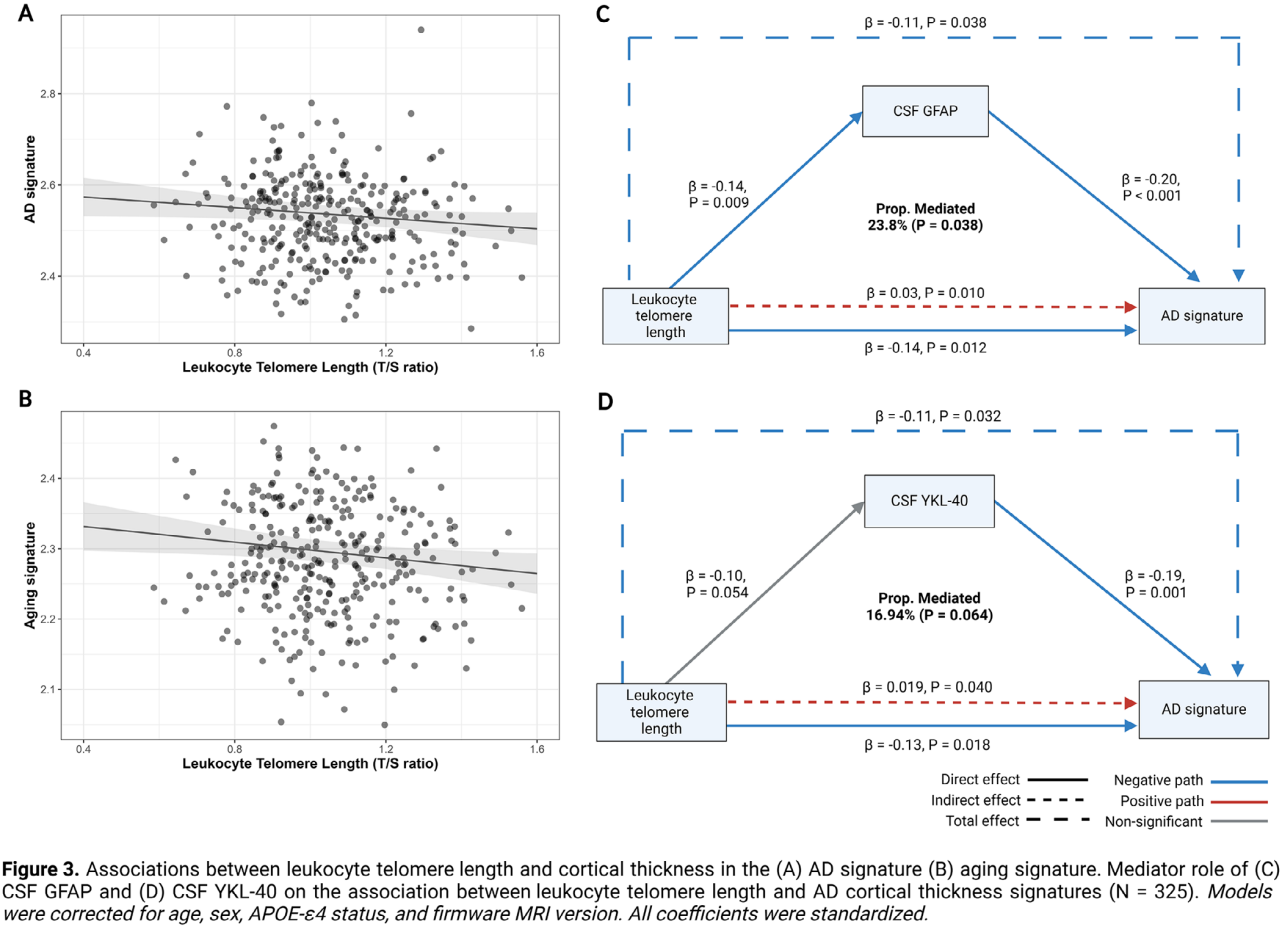# Shorter leukocyte telomere length is associated with distinct CSF biomarker dynamics across early AD stages in at‐risk individuals

**DOI:** 10.1002/alz70856_098828

**Published:** 2025-12-24

**Authors:** Blanca Rodríguez‐Fernández, Armand González Escalante, Patricia Genius, Tavia E Evans, Paula Ortiz‐Romero, Carolina Minguillón, Gwendlyn Kollmorgen, Nicholas J. Ashton, Henrik Zetterberg, Kaj Blennow, Juan Domingo Gispert, Arcadi Navarro, Marc Suarez‐Calvet, Aleix Sala‐Vila, Marta Crous‐Bou, Natalia Vilor‐Tejedor

**Affiliations:** ^1^ Barcelonaβeta Brain Research Center (BBRC), Pasqual Maragall Foundation, Barcelona, Spain; ^2^ Centre for Genomic Regulation (CRG), Barcelona Institute of Science and Technology (BIST), Barcelona, Spain; ^3^ Universitat Autònoma de Barcelona, Barcelona, Barcelona, Spain; ^4^ Hospital del Mar Research Institute (IMIM), Barcelona, Spain; ^5^ Universitat Pompeu Fabra, Barcelona, Spain; ^6^ Hospital del Mar Research Institute, Barcelona, Spain; ^7^ University of Vic‐Central University of Catalonia, Vic, Spain; ^8^ Erasmus MC, Netherlands, Rotterdam, Netherlands; ^9^ Trinity College Dublin Ireland, Dublin, Ireland; ^10^ Instituto de Salud Carlos III, Madrid, Spain; ^11^ Roche Diagnostics GmbH, Penzberg, Germany; ^12^ Department of Psychiatry and Neurochemistry, Institute of Neuroscience and Physiology, The Sahlgrenska Academy, University of Gothenburg, Mölndal, Gothenburg, Sweden; ^13^ King's College London, Institute of Psychiatry, Psychology & Neuroscience, Maurice Wohl Clinical Neuroscience Institute, London, United Kingdom; ^14^ Wallenberg Centre for Molecular and Translational Medicine, University of Gothenburg, Gothenburg, Sweden; ^15^ NIHR Biomedical Research Centre for Mental Health and Biomedical Research Unit for Dementia at South London and Maudsley, NHS Foundation, London, United Kingdom; ^16^ Hong Kong Center for Neurodegenerative Diseases, Hong Kong, Science Park, China; ^17^ Department of Neurodegenerative Disease, UCL Institute of Neurology, Queen Square, London, United Kingdom; ^18^ Wisconsin Alzheimer's Disease Research Center, University of Wisconsin‐Madison, School of Medicine and Public Health, Madison, WI, USA; ^19^ Clinical Neurochemistry Laboratory, Sahlgrenska University Hospital, Gothenburg, Sweden; ^20^ UK Dementia Research Institute at UCL, London, United Kingdom; ^21^ Department of Psychiatry and Neurochemistry, Institute of Neuroscience and Physiology, The Sahlgrenska Academy, University of Gothenburg, Mölndal, Sweden; ^22^ Institute of Neuroscience and Physiology, Department of Psychiatry and Neurochemistry, The Sahlgrenska Academy at University of Gothenburg, Mölndal, Sweden; ^23^ Paris Brain Institute, ICM, Pitié‐Salpêtrière Hospital, Sorbonne University, Paris, France; ^24^ Neurodegenerative Disorder Research Center, Institute on Aging and Brain Disorders, University of Science and Technology of China and First Affiliated Hospital of USTC, Heifei, China; ^25^ Clinical Neurochemistry Laboratory, Sahlgrenska University Hospital, Mölndal, Sweden; ^26^ Centro de Investigación Biomédica en Red de Bioingeniería, Biomateriales y Nanomedicina (CIBER‐BBN), Madrid, Spain; ^27^ Spanish National Center for Cardiovascular Research (CNIC), Madrid, Spain; ^28^ Institute of Evolutionary Biology (UPF‐CSIC), Barcelona, Spain; ^29^ BarcelonaBeta Brain Research Center (BBRC), Barcelona, Spain; ^30^ Institució Catalana de Recerca i Estudis Avançats (ICREA), Barcelona, Spain; ^31^ Servei de Neurologia, Hospital del Mar, Barcelona, Spain; ^32^ Fatty Acid Research Institute, Sioux Falls, SD, USA; ^33^ Department of Epidemiology, Harvard TH Chan School of Public Health, Boston, MA, USA; ^34^ Catalan Institute of Oncology (ICO)‐Bellvitge Biomedical Research Center (IDIBELL), Hospitalet de Llobregat, Spain; ^35^ Department of Genetics, Radboud Medical University Center, Nijmegen, Netherlands

## Abstract

**Background:**

Shorter telomeres are a hallmark of biological aging and have been associated with an increased risk of Alzheimer's disease (AD). However, their role in AD pathophysiology remains unclear. This study investigates the relationship between telomere length (TL), longitudinal cerebrospinal fluid (CSF) AD biomarkers, and brain structure at early stages of the AD *continuum*.

**Method:**

We included 346 cognitively unimpaired participants from the ALFA+ cohort, aged 49–71 years, at increased risk for AD (53.2% *APOE*‐ε4 carriers, 34% CSF amyloid‐positive). Participants had available CSF biomarker data, structural MRI, and leukocyte TL (LTL) measured by qPCR. AD‐related CSF biomarkers were assessed at baseline and longitudinally after a mean follow‐up of 3.45 years (SD = 0.58) with Elecsys® and NeuroToolKit CSF immunoassays (Roche Diagnostics International Ltd). Brain signatures were derived from the average cortical thickness in aging and AD‐vulnerable regions. Stratified models by *APOE*‐ε4 status and CSF amyloid‐tau (AT) classification were computed. Linear structural equation modeling was applied to assess the mediating role of CSF biomarkers in the association between LTL and cortical thickness.

**Result:**

Shorter LTL was associated with higher astrocytic reactivity and with longitudinal synaptic dysfunction increases. In *APOE*‐ε4 carriers and AT‐positive individuals, shorter LTL was associated with higher *p*‐tau181 and neurodegeneration markers [Figure 1]. *APOE*‐ε4 carriership modified the association between LTL and CSF YKL‐40 and longitudinal CSF sTREM2 changes over time [Figure 2]. Additionally, shorter LTL was associated with thicker cortex in aging and AD‐vulnerable regions [Figure 3A‐B], with astrocytic reactivity biomarkers partially mediating this association [Figure 3C‐D].

**Conclusion:**

These findings suggest that shorter telomeres may contribute to early AD progression, potentially through effects on astrocytic reactivity and brain structure.